# Dielectric Spectroscopy of Calcium Titanate Processed by Spark Plasma Sintering

**DOI:** 10.3390/ma16030975

**Published:** 2023-01-20

**Authors:** Pavel Ctibor, Josef Sedláček, Libor Straka, František Lukáč, Karel Neufuss

**Affiliations:** 1 Institute of Plasma Physics of the Czech Academy of Sciences, Za Slovankou 1782/3, 182 00 Prague, Czech Republic; 2 Faculty of Electrical Engineering, Czech Technical University, Technicka 2, 166 27 Prague, Czech Republic

**Keywords:** calcium titanate, spark plasma sintering, permittivity, dielectrics, band gap

## Abstract

Calcium titanate (CaTiO_3_) powder was compacted by spark plasma sintering (SPS). The resulting products were subjected to the phase stability study and dielectric characterization. The change in temperature of SPS between 1100 °C and 1250 °C had a clear and straightforward effect on density, porosity, relative permittivity, loss tangent, and DC resistivity. Since the SPS itself introduces certain oxygen deficiency into Ti-perovskites, all samples were annealed after SPS. However, this post-processing did not mask the effects of the SPS regime. Optical reflectance measurements were completed to compare and quantify the sample coloration and support the dielectric results with corresponding optical band gap estimations. Subtle changes in the CaTiO_3_ crystal lattice arrangement, completed between 1150 °C and 1250 °C and documented in the literature for conventionally sintered samples, could not be confirmed for SPS-prepared calcium titanate. The novelty of this research work is in producing very stable dielectric ceramics and an indication of the SPS processing parameters suitable for this. The best sample showed at 1 MHz frequency the combination of relative permittivity 370, loss tangent 0.008, and DC resistivity 3 × 10^12^ Ωm.

## 1. Introduction

Calcium titanate (CaTiO_3_) with a perovskite structure is a ceramic material often exploited in the fabrication of electronic devices. A powder neutron diffraction study [1] suggested that there may be as many as three phase transitions in CaTiO_3_: (i) from cubic to body-centered tetragonal at 1580 K (i.e., 1307 °C), (ii) to a possible centered orthorhombic phase at 1500 K (i.e., 1227 °C), and (iii) to the low temperature primitive orthorhombic phase at 1380 K (i.e., 1107 °C). All of the phases are related to the perovskite structure via small distortions of ions from their ideal perovskite positions. Between 1150 °C and 1250 °C the tetragonal phase exists [2]. However, the tetragonal phase appears metrically cubic within the resolution of the diffractometer [2].

Calcium titanate has been produced using soft chemistry such as sol–gel, hydrothermal, and coprecipitation methods [3,4,5,6]. Monolithic ceramics can be densified by sintering, which is a technique that influences the microstructures and properties of materials. The general concept of sintering is an interface elimination process by atomic transport that reduces high surface energy. In this way, the total free energy of the system is reduced [7]. The conventional sintering procedure applies a constant heating rate in a furnace with a holding time at the stabilized sintering temperature [8].

Spark plasma sintering (SPS) enables the very rapid fabrication of bulk ceramic materials. It is a consolidation technique combining pulsed electric currents with uniaxial pressure-induced compaction. Heating rates, applied pressures, and pulsed current patterns are the main factors responsible for the enhancement of densification kinetics and conservation of the fine-grained structure of materials [9]. The SPS was used for the processing of dielectric ceramics, although it brought a structural disorder [10] that affected also dielectric properties [9,11]. The SPS process is fast and thanks to it a grain over-growth was successfully suppressed in dielectric ceramics [8]. Applying pressure, together with an appropriate thermal cycle, one can obtain fully dense ceramics with grain sizes as fine as between 50 and 100 nm [12].

There are some microstructural aspects of CaTiO_3_ associated with specific phase compositions. Pramanik et al. [13] sintered CaTiO_3_ by SPS at 1200 °C temperature, 50 MPa pressure, and 5 min dwell time. Orthorhombic to tetragonal transformation manifested itself by twins. Riaz et al. [14] consolidated the material by SPS at a markedly lower temperature, 1025 °C, whereas pressure was 76 MPa and dwell time 5 min. In the experiment, the vacuum level was at 1 mbar and the cooling rate was 208 °C/min. They indicated an orthorhombic (Pbnm) phase of CaTiO_3_ with a crystallite size of 200 nm.

Calcium titanate dielectrics were characterized by our team in the past as synthesized via plasma spraying—the samples were measured in the as-sprayed form [15] as well as in variously annealed forms [16]. In this research, the material was documented as very resistant to chemical and phase changes induced by the rapid heating–cooling cycles. The only effect regularly induced by the extremely rapid cooling was the oxygen deficiency associated with vacancies [16]. The evidence of the O deficiency was due to the coloration of samples—calcium titanate is light red after slow sintering but dark blue–grey after fast thermal processes. Oxygen-vacant positions in the lattice induced electronic imbalance and these centers served as absorption sites for the red (and partly white) component of photonic radiation. The resulting reflected light was therefore less intensive and blue-shifted. Annealing in a furnace was proven as the best way how to restore the stoichiometric oxygen content. After this experience, we decided to sinter also by SPS the CaTiO_3_ powder—this rather long-time known but still important dielectric ceramics. Our goal was to sinter via an economically advantageous procedure a good quality dielectric material, a prospective one from the standpoint of an optimum compromise solving the combination of rather contradictory criteria mentioned in the next paragraph.

The dielectric materials are commonly described in terms of relative permittivity, which parameter is derived from a measurable parameter called capacitance and corresponds to the physical quality of the material called polarization. The higher the polarization, the larger the energy stored in the material. Dielectric loss (expressed as the loss tangent) is a parameter referring to the dissipation of the energy to heat and its low value is desirable for each circuit component to stay cold and work efficiently. In the literature, two rather different dielectric behaviors of calcium titanate could be found for sintered samples. (i) Dubey et al. [17] reported high relative permittivity 490, however, with a high loss tangent of 1.006. A second sort (ii) is represented by works of Mousavi [18], with relative permittivity of only 190 and reasonably low loss tangent of 0.033 or Zhou et al. [19] reporting relative permittivity 190 and loss tangent only 0.003. To find a good compromise in terms of high relative permittivity and simultaneously low loss tangent represents a challenge for the sintering processes and would be the key aspect of our actual study.

## 2. Experimental 

### 2.1. Sample Preparation

The powder, originally dedicated to plasma spraying, described in [15], was made finer in size by a planetary mill Pulverisette 5 (Fritsch, Idar-Oberstein, Germany). The weight ratio was 1:10 CaTiO_3_ versus balls. The milling was performed in a tungsten carbide vial with tungsten carbide balls (diameter 10 mm) under air atmosphere. Milling velocity was 240 rpm and the total milling time was 25 h. The laser particle size analyzer Mastersizer 3000 (Malvern, UK) was used for the measurement of the final powder size.

Sintering of the powder was carried out in an evacuated chamber of the SPS machine (10-4, Thermal Technology LLC, Minden, NV, USA). The powder was kept in a carbon die with pressure applied via two (the top one and bottom one) carbon pistons that were in-house machined from graphite blocks. Sigrafine R8710 (SGL carbon, Wiesbaden, Germany) blocks were used, characterized by compressive strength 170 MPa. The graphite die had internal diameter of 20 mm and external one of 70 mm. The pistons had diameter of 19.9 mm and length of 40 mm. The space between piston cylindrical surface and the die was tightened with graphite foil. Without this tightening, the finest powder particles could fill the spacing and make the SPS parts disassembling problematic, with a risk of sample destruction at this step. Flat surfaces of the pistons were separated from the sample with the same foil to prevent surface roughening of the piston. For the prevention of carbon contamination of the sample, this arrangement is usually not enough, and a tungsten foil must be applied. In the case of CaTiO_3_ instead of them, several tens of millimeters of the sample thickness were removed from both sides via grinding after SPS firing. The main factors influencing the SPS sample character are maximum temperature, uniaxial pressure, dwell time, and heating/cooling ramps. Cooling was performed at the rate dictated by the furnace’s thermal inertia. In case of the actual study, only the temperature was varied, and other parameters were kept constant.

All samples were annealed in air after sintering at the atmospheric pressure for 1 h at a temperature of 1000 °C in order to reoxidize the structure reduced after SPS processing. Samples were annealed in the furnace all together in the same heat cycle: 8 °C/min heating and cooling. A programmable laboratory furnace equipped with SiC-based heaters (Clasic, Czech Republic) was employed for the experiment. This additional step makes the SPS manufacturing of samples less economical, but the annealing dwell time is substantially shorter than the conventional sintering dwell time.

The produced samples were cylinders with 20 mm in diameter and 4 to 5 mm in height. We produced four samples labeled according to the temperature [°C] 1100, 1150, 1200, and 1250. Sample 1150 was replicated also in a nitrogen atmosphere kept at 200 Pa pressure (label 1150N2). The reason for nitrogen application was, based on our experience, that for many titanates vacuum, plus the carbon-containing SPS parts, represent the too reducing environment and the result is not only existence of oxygen vacancies in the sintered compacts but also strong contamination of the samples by carbon. Nitrogen gives chance to obtain O deficiency only, without C-admixing.

The set of temperatures was selected because this is the range of existence of important phase changes in CaTiO_3_. Additionally, we estimated that even higher temperatures would cause too strong oxygen deficiency and carbon contamination of samples. 

### 2.2. Characterization Techniques

#### 2.2.1. Porosity and Microstructure 

The sintered density and open porosity of the calcium titanate ceramics were measured according to Archimedes’ principle, and were calculated using the Equation (1) [20],

D_sint_ = W_0_/(W_2_ − W_1_) × ρ_wat_ (1)

where D_sint_ is the sintered density, W_0_, W_1_, and W_2_ are the dry weight, weight in water of the water-saturated specimen, and weight in air of the water-saturated specimen, respectively, obtained using a high-precision electronic balance. In addition, ρ_wat_ is the density of water (1.0 g/cm^3^). Total porosity, which must include also the closed porosity, and needs different measurement techniques, was not classified.

The surface of specimens was smoothened by grinding to eliminate the as-sintered surface roughness and possible superficial contamination from the carbon foil separators used at SPS. For the microstructural observation, fractured surfaces were utilized, to reveal the interior of the samples. Scanning electron microscopy (SEM) observation was performed using Phenom-Pro microscope (Thermo Fisher Sci., Breda, The Netherlands) equipped with CeB_6_ thermionic cathode and working in backscattered electron (BSE) mode. The images were collected at 10 kV electron beam voltage. 

#### 2.2.2. Phase and Chemical Composition 

Phase compositions of samples were determined by powder X-ray diffraction (PXRD) methods. The measurements were carried out on vertical powder θ-θ diffractometer D8 Discover (Bruker AXS, Karlsruhe, Germany) using Cu Kα radiation with Ni Kβ filter. Diffracted beam was detected by 1D detector LynxEye. Bragg–Brentano geometry was employed with 0.5° fixed divergent slit in the primary beam. In situ annealing in air was performed in a high-temperature chamber MRI (Bruker AXS, Germany) using platinum holder with welded thermocouple in the measured area. The angular range was from 20 to 100 °2θ, step size 0.03 °2θ and the total time in each step was 192 s. Phase identification was completed using X’Pert HighScore program which accessed PDF-4+ database of crystalline phases. Quantitative Rietveld refinement was performed in TOPAS V5, aiming at determination of wt.% of all the identified phases following the theory from publications [21,22]. Lattice parameters a, b, and c were quantified. Small texture correction was included in order to improve the intensities of reflections according to the March–Dollase approach [23].

The X-ray fluorescence (XRF) of powder was completed using energy-dispersive (EDXRF) spectrometer S2 PUMA (Bruker, Leipzig, Germany) equipped with HighSense LE SDD detector.

#### 2.2.3. Dielectric Parameters

Dielectric parameters of the ceramic compacts were measured on cylindrical sample geometry forming a monolayer capacitor. Faces of the sample were covered with aluminum in an evaporating apparatus. Before them, samples were grinded by the SiC paper to eliminate surface unevenness. With using a mask, a three-electrode system was applied to diminish the stray current effect during the measurement. One face was Al-coated completely and the second one with an internal circle electrode, 12 mm in diameter, and external ring electrode (earth-connected during the measurements), separated from the internal ring with a 1 mm wide gap.

The electric field was applied along the pressure direction (i.e., contacts made on the cylinder face). The capacitance of the samples was measured using a programmable impedance analyzer model 4284A (Agilent, Santa Clara, CA, USA), while the sample was clamped using a high precision sample fixture 16451B (Agilent, USA). 

The dielectric constant (i.e., relative permittivity) was calculated according to the Equation (2) [18],

ε_r_ = C × d/ε_0_ × A (2)

where ε_r_ is the relative permittivity, C (F) is the equivalent parallel capacitance obtained from the measurement, d (m) is the thickness of the sample, A is the surface electrode area of the internal circle, and ε_0_ is the permittivity of vacuum (8.85 × 10^−12^ F/m). The dielectric loss obtained in form of the value of the loss tangent tan δ was measured simultaneously with the relative permittivity [24].

The DC volume resistance was measured on the same samples as for capacitance, using Keithley 6517-B high resistance resistometer. Applied voltage was set to 50 ± 0.2 V. Customized Keithley 6104 shielded test enclosure was used in order to avoid errors from any external noise. The resistivity ρ was calculated from measured resistance and specimen dimensions using the equation

**Figure 1:**
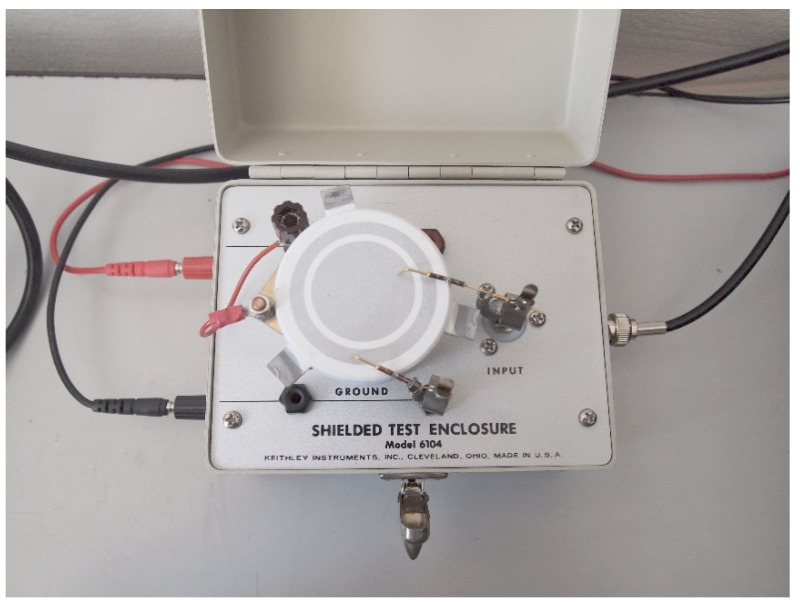
Customized Keithley 6104 shielded test enclosure.

ρ = (R·A)/d (3)

where R (Ω) is sample electrical resistance, A (m^2^) is area of the internal circle electrode and d (m) is the sample thickness. The enclosure is displayed in Figure 1 (with a different sample, having a much larger diameter, 60 mm).

#### 2.2.4. Reflectance and Band Gap

The optical diffuse reflectance spectroscopy was completed using a UV–vis–NIR scanning spectrophotometer MPC 3100 (Shimadzu, Kyoto, Japan) with a multi-purpose large sample compartment. The reflectance curves were obtained between 200 nm and 2000 nm. The reflectance standard was introduced, which is a BaSO_4_ mirror with 100% reflection in the studied range.

The optical band gap energy can be computed using the Tauc relationship [25]:

(α × hν)^n^ = A × (hν − E_g_) (4)

with the absorption coefficient α, the photon energy hν, a constant A, and the optical band gap E_g_. The exponent n denotes the nature of the transition; its value is 2 for direct allowed transition and 1/2 for indirect allowed transition. Knowing that CaTiO_3_ compound have a direct transition [20,21], the n exponent value is 2:

(α × hν)^2^ = A × (hν − E_g_) (5)

On the other hand, α is related to the Kubelka–Munk function F:

*F*(R) = (1 − R)^2^/2R = α/S (6)

where R is the reflectance and S is the scattering factor. E_g_ [eV] is determined from the extrapolation of the linear part of the Tauc plot up to the intercept of the energy x-axis.

## 3. Results and Discussion

### 3.1. Powder and Its Processing

**Figure 2:**
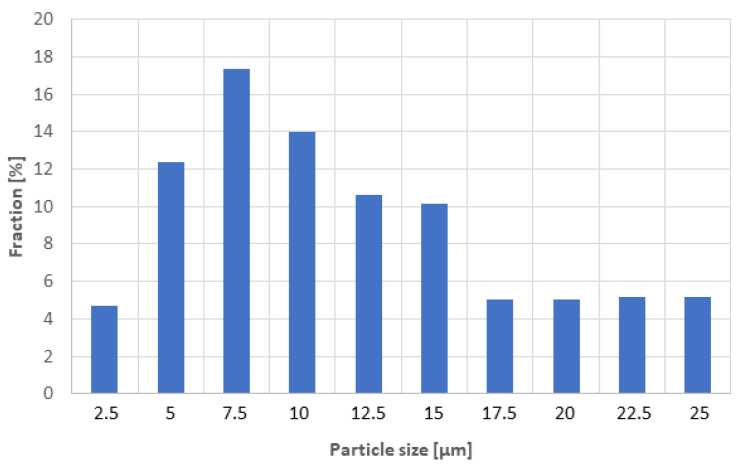
Particle size distribution of the starting powder.

The starting coarse powder was according to the XRD analysis composed solely of calcium titanate, PDF card #00-022-0153, the details will be presented below. According to XRF analysis, the recalculated content of the basic oxides showed also about 0.5 wt.% of each impurity: ZrO_2_, SiO_2_, La_2_O_3_. The final particle size distribution is in Figure 2. The maximum of particles was between 7 and 8 µm, but on the other hand, the remaining up to 20% of the particles had a size still over 15 µm.

### 3.2. SPS Processing Parameters

**Figure 3:**
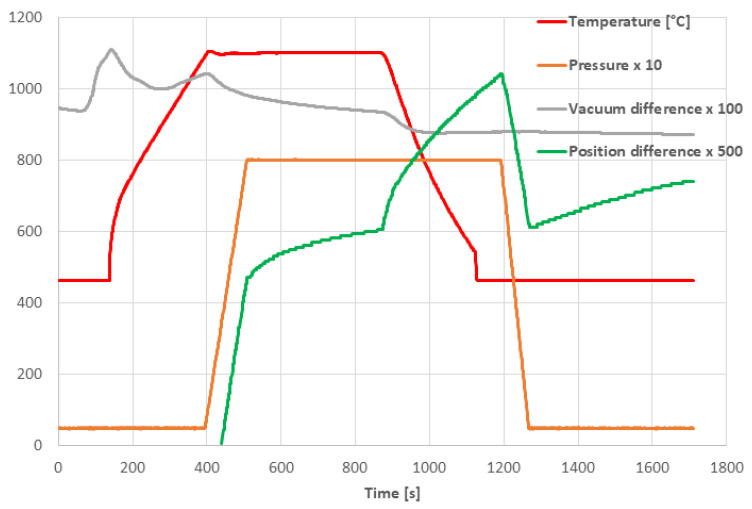
Time dependence of selected SPS parameters for the sample 1100-80-8 min: Temperature (°C), Pressure (MPa), Vacuum difference (Pa), Position difference (mm) (scales are multiplied, as indicated).

The SPS cycle is displayed in Figure 3. The temperature shown here was monitored with an optical pyrometer. For this purpose, a hole was drilled in the die from the side and the die wall thickness at the sample side was only 3 mm. The uniaxial pressure was switched on at the moment of reaching the maximum temperature of 1100 °C and was stabilized at its maximum value of 80 MPa after 200 s from the beginning, Figure 3. As a consequence of the pressure application, the position of the upper piston was shifted down, i.e., the sample height was decreased because of powder compression. This is, in the graph, expressed as the Position difference (compression is oriented up). There are two contractions of the sample—the first one after the uniaxial pressure application and the second one after the start of the cooling. This last one should be the rest of the compaction process plus the thermal shrinkage of the material and represents about 25% of the final sample height. This inverse thermal expansion was later partly compensated after the uniaxial pressure release. The evacuation in the chamber was worsened (i.e., “Vacuum difference” oriented up) at two moments during the SPS cycle. The first time during the initial heating, because of moisture extraction, and the second time after the beginning of the uniaxial pressure application because of air release from the spaces between the powder particles. The duration of the entire SPS process, including cooling, was about 20 min, Figure 3. For the other samples, only the temperature is changed but all other features of the sintering schedule remained the same.

**Figure 4:**
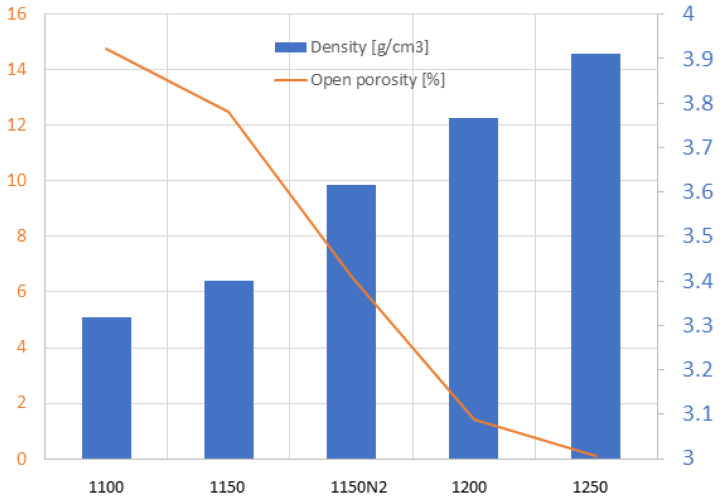
Open porosity (line) and density of the samples (columns).

As Figure 4 shows, with increasing sintering temperature, the density increased, and open porosity decreased. The lowest open porosity, detected in sample 1250, was only 0.1%. This sample manifested 98.3% of the theoretical density of CaTiO_3_ (which is 3.98 g/cm^3^). The sample sintered in nitrogen (at 1150 °C) exhibited a density of 3.62 g/cm^3^ and an open porosity of 6.5%. Its values are better compared to sample 1150. The densification could be attributed to both grain boundary diffusion and grain boundary migration [7]. 

### 3.3. Phase Analysis and Microstructure

Considering the literature based on conventionally prepared CaTiO_3_ [1,2], we produced two (theoretically) orthorhombic samples (i.e., 1100 and 1150), one sample just transforming to tetragonal (1150-nitrogen; protective atmosphere should shift the transformation temperature slightly down), one tetragonal sample (1200) and one sample just transforming to cubic phase (1250). Nitrogen gives us a chance to obtain O deficiency only in the dedicated sample, without carbon admixing [26].

According to the XRD measurements, the orthorhombic Pbnm cell was detected in all SPS samples. There is necessary to mention that all SPS samples contain also a few percent of impurity phases (i.e., CaTiO_3_ precursors): TiO_2_ rutile and CaCO_3_ calcite. The highest content of TiO_2_, 0.33 wt.%, was detected in sample 1250 and calcite was the highest concentration 0.88 wt.%, detected in sample 1100. The sample sintered at 1250 °C contained also 5.86% of carbon (graphite), Table 1, which was contaminated by the SPS device parts. The densification of this sample was fully in accordance with the trend followed by all other samples, but due to high temperature, carbon contamination was significant. The lattice parameters are summarized in Table 2. The lattice expansion (the unit cell volume) was maximal at 1150 °C.

**Figure 5:**
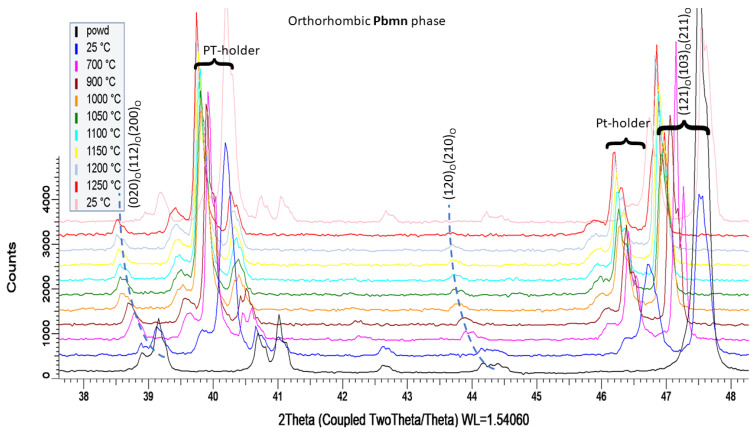
In situ temperature X-ray diffraction of the powder up to 1250 °C. The dashed lines added for a better eye guidance.

The starting powder, analyzed by XRD in situ during heating up to 1250 °C, included at all temperatures orthorhombic Pbnm phase, Figure 5. The indication of its presence is the peak “(120)(210)” (where the subscript “O” means orthorhombic), which was shifted at high temperatures to lower angles due to the thermal expansion but did not disappear. The peaks corresponding to the platinum sample holder are indicated in the Figure 5. Based on this HT-XRD we produced SPS non-tetragonal samples also at 1200 and 1250 °C. Thanks to this fact, the internal stress in our SPS samples 1200 and 1250 is lower, because of the absence of the transformations, and this would be advantageous for the dielectric behavior. Concerning the physical reasons for the transformation, most probably, the presence of impurities in the powder and its pre-sintering thermal history were the key factors.

**Figure 6:**
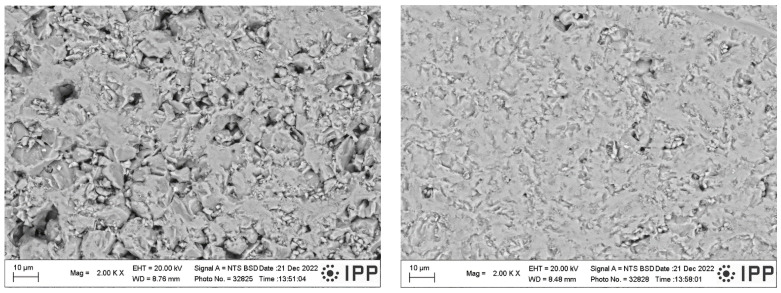
Fracture surface of the samples 1100 (**left**) and 1250 (**right**), SEM-BSE.

Fracture surfaces of the samples 1100 and 1250 (i.e., the low and high SPS temperature extremes) are shown in Figure 6. The open porosity of these samples was 14.5% and 0.1%, respectively (Figure 4). The SEM micrographs document a certain difference in porosity and in material cohesion; sample 1100 exhibits more grain boundaries and weaker inter-grain contact. For both samples, the grain size is about 10 µm or slightly less (visual observation and using a scale bar). This size correlates roughly with the average of the powder size distribution, Figure 2. The presence of ultrafine particles was detected in both samples. These particles adhere well to the coarser crystals. We believe that the cause of these particles is the fragility of the powder during the milling process, while the SPS was too fast to ensure full sintering of these particles. Graphite serving as an intrinsic lubricant, and also preventing purely intercrystalline fracture, was dispersed in the whole mass of sample 1250. For the porosity formation, there could be partly different reasons: for sample 1100, simply the thermal energy was not high enough to ensure full densification, whereas, in sample 1250, the vacancy clustering [27] could play a role.

### 3.4. Dielectric Properties

**Figure 7:**
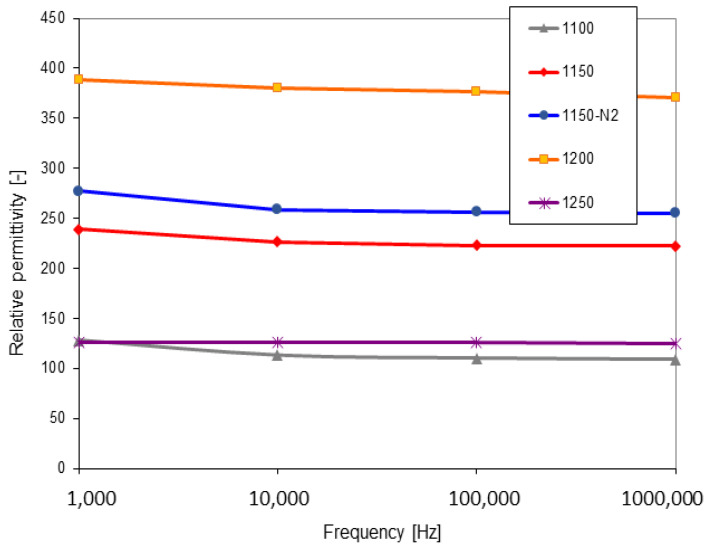
Relative permittivity as a function of frequency.

**Figure 8:**
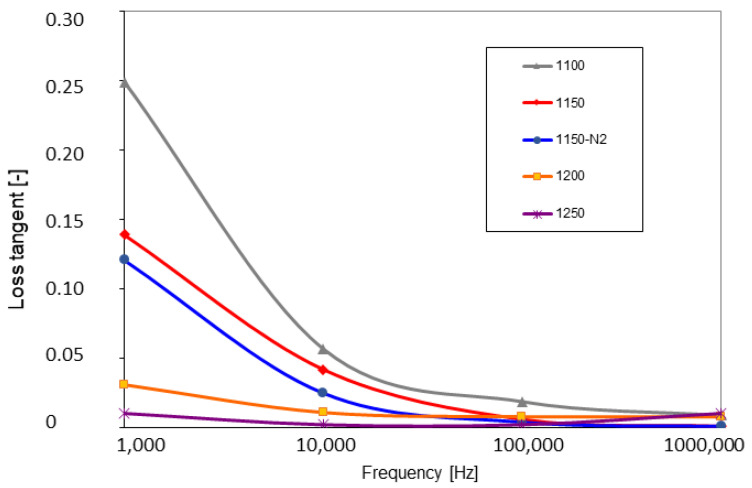
Loss tangent as a function of frequency.

**Figure 9:**
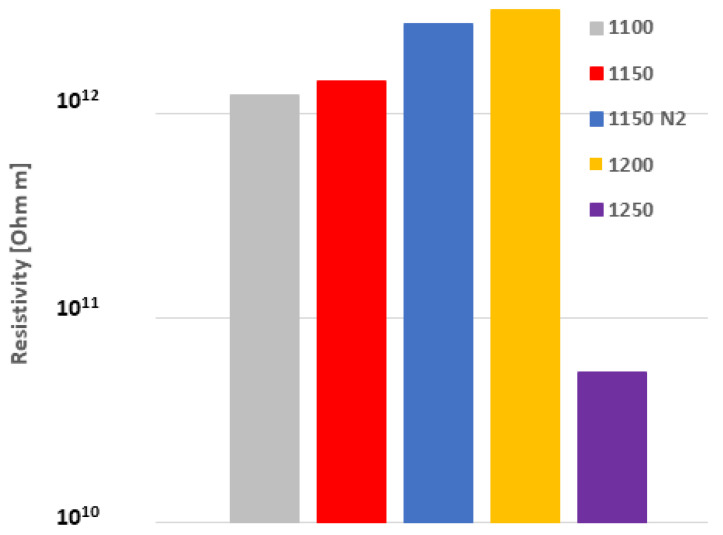
DC resistivity. Increasing SPS temperature from the left side.

The higher the temperature of SPS, the higher the relative permittivity, Figure 7. The temperature of 1250 °C was the only exception with a substantial decrease in permittivity. Since the permittivity, losses, and resistivity are strongly connected together, the explanations for the permittivity decrease in the 1250 sample will be found below. The permittivity was very stable for all samples versus the changing frequency. The loss tangent had also a clear trend, here without any exception, Figure 8. The high SPS temperature led to its stabilization, whereas the low temperature led to a pronounced loss tangent increase at low frequency. Concerning the DC resistivity, Figure 9, the high SPS temperature also increased its value. Sample 1250, as an exception, exhibited resistivity of only about 7 × 10^10^ Ωm, whereas the most resistive sample 1200 had over 3 × 10^12^ Ωm. This disadvantage of the 1250 sample was caused by free carbon, which is conductive. 

The best among samples in the combination of the relative permittivity and loss tangent was the one sintered at 1200 °C. However, the cubic phase, announced in some publications [1,2] around this temperature, was not found in our samples via PXRD.

These results are consistent with the fact that the charge polarization (or relaxation polarization) of simply processed structurally dense materials with fewer pores produced considerably larger relative permittivity at low frequencies. In addition, at 200 kHz, the dielectric loss of calcium titanate ceramics sintered at different sintering conditions was similar and low, ranging from 0.02 to 0.06.

A furnace-sintered calcium titanate [18] exhibited a relative permittivity of 190 and a loss tangent of 0.033. Calcium titanate sintered from raw materials, including waste duck eggshell, exhibited a relative permittivity of 78 and a loss tangent of 0.02 at a density of 3.65 g/cm^3^ (i.e., 91.7% of theoretical density) and porosity 0.54% [28]. However, in that case, the sintering needed 3 h to dwell at 1350 °C. In our case, higher density was received at lower temperatures, 1200 and 1250 °C. 

An even more promising dielectric was calcium titanate synthesized by a conventional solid-state reaction, with relative permittivity of 312 and a loss tangent of 0.02 [29]. Our best sample showed a combination of relative permittivity of about 370 with a loss tangent of about 0.008. All these values are valid for 1 MHz frequency.

Based on our experiments, we can say that the behavior (for the concept of i and ii please return to the Introduction) (i) means some conductive component in the microstructure because the loss tangent is extremely high. Behavior (ii) represents a well-balanced compromise between a rather high permittivity and low loss. In our case, the relative permittivity was even higher than the literature values and stable versus changing frequency, a fact which is also important. This should be connected with the lowered band gap. Explanations have been provided in the following paragraph of this study. 

### 3.5. Reflectance and Optical Band Gap

**Figure 10:**
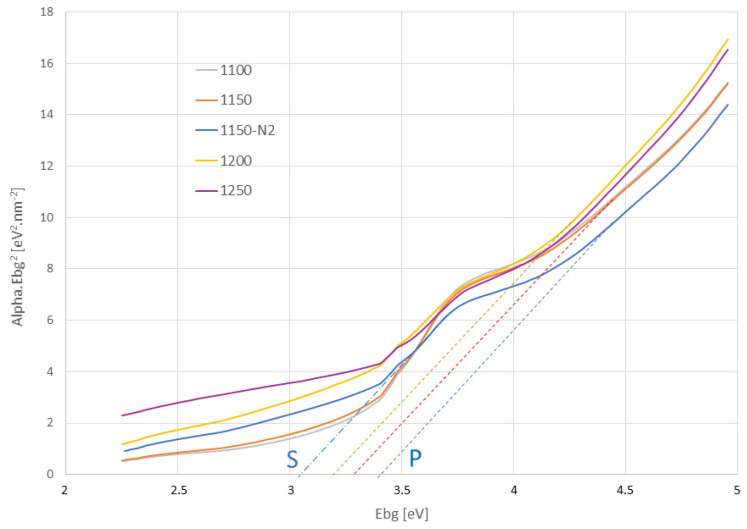
Optical band gap energy estimation for all samples.

Band gap energy E_bg_, based on optical diffuse reflectance spectroscopy, Figure 10, was estimated assuming direct electron transition from the valence band (VB) to the conduction band (CB). The intersections of prolonged curve branches with the X-axis show the estimated energies. The SPS samples had E_bg_ between 3.25 eV for sample 1200 (and also sample 1250; the dashed lines), and 3.40 eV for the sample sintered in nitrogen atmosphere 1150N2.

All these values stand for the primary gap, denoted P in the graph. The value 3.40 eV was listed for a single crystal [30] and also for the furnace-sintered polycrystalline CaTiO_3_ [31]. As high E_bg_ as 3.51 eV was reported for polymeric precursor-based CaTiO_3_ powder [32]. The secondary gap denoted “S” in the graph, of the nitrogen atmosphere sintered 1150N2 sample was localized at about 3.0 eV. Note please, that only for this sample, blue color, is the secondary gap indicated in the Figure, for better clarity, although also in other samples it exists. One of the features, responsible for the existence of such a secondary gap, could be a partly amorphous structure or the presence of impurities. This secondary gap, called also the Urbach tail, is attributed to the presence of localized electronic states near the band edges of amorphous or nanocrystalline semiconductors [33]. 

The graph, Figure 10, shows that optoelectronic properties (the primary gap) of the SPS samples were compared with the conventionally prepared CaTiO_3_, c.f. the literature mentioned above [30,31,32]. The electron energetic states forming the tail (i.e., at about 3 eV) could be the shallow donor levels induced by H-protons within the bandgap in titanates [34]. All interfaces exhibit a large number of traps, which form a trap band with a typically much lower band gap (up to 0.4 eV decrease) [35]. This was also the case of our SPS samples. The different band gap values indicate the existence of various intermediary levels between the CB and VB, which were induced by a structural disorder. Thus, a decrease in the bandgap can be attributed to defects, such as distortions along Ca-O linkages, and moreover, to the presence of the TiO_2_ secondary phase [32]. Radiative recombination between trapped electrons and trapped holes in tail states or gap states is possible in the CaTiO_3_ when it exhibits the tail states in the band gap [36]. The calculated band gap of amorphous CaTiO_3_ was only 2.22 eV, so for example the partly crystalline CaTiO_3_ could exhibit values in a wide range between 2.22 eV and 3.48 eV [36]. The symmetry of the CaTiO_3_ system is modified intrinsically by the random movement of the calcium, due to its ionic character. The same phenomena, dangling bonds and H-protons within the band gap, could be responsible also for the enhanced dielectric loss tangent at low frequency, present in the SPS samples.

**Figure 11:**
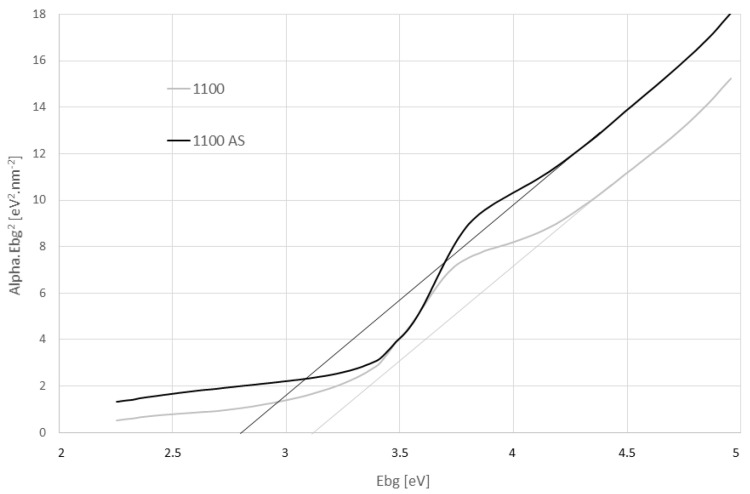
Optical band gap energy estimation for sample 1150 and its as-sintered “AS” analog.

Figure 11 shows the optical band gap energy estimation for sample 1150 and its non-annealed (as-sintered, “AS”) analog. The disappearance of some intermediate levels, due to the reduction of the structural disorder via annealing, was recorded [37]. The increase in band gap after annealing (the thin lines; E_bg_ > 3 eV versus the E_bg_ < 3 eV for the as-sintered) can be ascribed to the reduction in defects or impurities that give rise to intermediary energy levels in the band gap region of a disordered material [37].

The thermal post-processing did not mask fully the effects of the SPS regime. In another case, there would be detected any secondary gap, any tail states, and the calcium titanate bulk compacts would exhibit a rather high bandgap. Such “high-bandgap CaTiO_3_” could show lower relative permittivity and possibly also even lower loss tangents. 

## 4. Conclusions

Calcium titanate (CaTiO_3_) powder was compacted by spark plasma sintering (SPS). All samples were annealed after SPS. The temperature of SPS between 1100 and 1250 °C had a clear and straightforward effect on density, porosity, relative permittivity, loss tangent, and DC electric resistivity. Density increased with the temperature of SPS whereas open porosity decreased. The relative permittivity increased with SPS temperature. The loss tangent decreased with SPS temperature and became less frequency-dependent. The DC resistivity increased with the SPS processing temperature. The only exception from the above trends was the sample sintered at 1250 °C, where free carbon, coming from the SPS equipment, was detected as the contaminant.

Subtle changes in the CaTiO_3_ crystal lattice arrangement, taken place between 1150 and 1250 °C, and documented in the literature for conventionally sintered samples, could not be confirmed for SPS-processed calcium titanate. The transformation temperatures, if in existence, are shifted up, because the ultra-fast heating conserved the room-temperature Pbnm phase. Such conservation is possible with a help of the impurities in the starting powder.

The novelty of this study is in producing very stable dielectric ceramics and an indication of the SPS processing parameters suitable for its production. The best sample, sintered at 1200 °C, showed at 1 MHz the combination of relative permittivity 370, loss tangent 0.008, and DC resistivity 3 × 10^12^ Ωm, overperforming sintered CaTiO_3_ materials reported earlier.

To obtain this combination of dielectric parameters, we could recommend doing the powder processing, in our case: producing CaTiO_3_ bricks and crushing them down to fine powder instead of performing SPS directly with in situ reacting precursors of CaTiO_3_, such as calcium carbonate and titanium dioxide are. 

## Figures and Tables

**Table 1:** Content of phases in wt.% (based on XRD).

Sample	CaTiO_3_	TiO_2_	CaCO_3_	C
1100	98.98	0.14	0.88	0
1150	99.28	0.15	0.57	0
1150-N2	99.03	0.13	0.84	0
1200	99.50	0.11	0.39	0
1250	93.16	0.33	0.65	5.86

**Table 2:** Lattice parameters (in Angströms) as a function of SPS temperature.

Sample	a	b	c
1100	5.44313	7.64322	5.38133
1150	5.44397	7.64443	5.38159
1200	5.44345	7.64503	5.38076
1250	5.44333	7.64473	5.38103

## Data Availability

The data serving for elaboration of this manuscript are non-public. They are stored by the authors and will be available to the readers upon email request ctibor@ipp.cas.cz.
